# Maintaining safe lung cancer surgery during the COVID-19 pandemic in a global city

**DOI:** 10.1016/j.eclinm.2021.101085

**Published:** 2021-08-20

**Authors:** Stephanie Fraser, Ralitsa Baranowski, Davide Patrini, Jay Nandi, May Al-Sahaf, Jeremy Smelt, Ross Hoffman, Gowthanan Santhirakumaran, Michelle Lee, Anuj Wali, Harvey Dickinson, Mehmood Jadoon, Karen Harrison-Phipps, Juliet King, John Pilling, Andrea Bille, Lawrence Okiror, Sasha Stamenkovic, David Waller, Henrietta Wilson, Simon Jordan, Sofina Begum, Silviu Buderi, Carol Tan, Ian Hunt, Paul Vaughan, Melanie Jenkins, Martin Hayward, David Lawrence, Emma Beddow, Vladimir Anikin, Aleksander Mani, Jonathan Finch, Hendramoorthy Maheswaran, Eric Lim, Tom Routledge, Kelvin Lau, Leanne Harling

**Affiliations:** a Department of Thoracic Surgery, Guy's Hospital, London, United Kingdom; b Department of Thoracic Surgery, St Bartholomew's Hospital, London, United Kingdom; c Department of Thoracic Surgery, University College London Hospital, London, United Kingdom; d Department of Thoracic Surgery, Hammersmith Hospital, London, United Kingdom; e Department of Thoracic Surgery, Royal Brompton Hospital, London, United Kingdom; f Department of Thoracic Surgery, Harefield Hospital, London, United Kingdom; g Department of Thoracic Surgery, St George's Hospital, London, United Kingdom; h South East London Cancer Alliance, United Kingdom; i Department of Surgery and Cancer, Imperial College London, United Kingdom

**Keywords:** SARS-CoV-2, Thoracic surgery, Lung Cancer

## Abstract

**Background:**

SARS-CoV-2 has challenged health service provision worldwide. This work evaluates safe surgical pathways and standard operating procedures implemented in the high volume, global city of London during the first wave of SARS-CoV-2 infection. We also assess the safety of minimally invasive surgery(MIS) for anatomical lung resection.

**Methods:**

This multicentre cohort study was conducted across all London thoracic surgical units, covering a catchment area of approximately 14.8 Million. A Pan-London Collaborative was created for data sharing and dissemination of protocols. All patients undergoing anatomical lung resection 1st March-1st June 2020 were included. Primary outcomes were SARS-CoV-2 infection, access to minimally invasive surgery, post-operative complication, length of intensive care and hospital stay (LOS), and death during follow up.

**Findings:**

352 patients underwent anatomical lung resection with a median age of 69 (IQR: 35–86) years. Self-isolation and pre-operative screening were implemented following the UK national lockdown. Pre-operative SARS-CoV-2 swabs were performed in 63.1% and CT imaging in 54.8%. 61.7% of cases were performed minimally invasively (MIS), compared to 59.9% pre pandemic. Median LOS was 6 days with a 30-day survival of 98.3% (comparable to a median LOS of 6 days and 30-day survival of 98.4% pre-pandemic). Significant complications developed in 7.3% of patients (Clavien-Dindo Grade 3–4) and 12 there were re-admissions(3.4%). Seven patients(2.0%) were diagnosed with SARS-CoV-2 infection, two of whom died (28.5%).

**Interpretation:**

SARS-CoV-2 infection significantly increases morbidity and mortality in patients undergoing elective anatomical pulmonary resection. However, surgery can be safely undertaken via open and MIS approaches at the peak of a viral pandemic if precautionary measures are implemented. High volume surgery should continue during further viral peaks to minimise health service burden and potential harm to cancer patients.

**Funding:**

This work did not receive funding.

**Research in context**

**Evidence before this study**

The SARS-CoV-2 pandemic has had a huge impact on surgical specialties across the UK, with significant reductions in elective surgical volume. The rationale for this is multifaceted, however includes lack of intensive care provision, redeployment of staff, repurposing of theatre space, risk of viral spread during aerosol generating procedures, and a fear of increased morbidity and mortality associated with SARS-CoV-2 infection in the perioperative period. Data surrounding the impact of SARS-CoV-2 on radically treatable lung cancer patients remains conflicting, with only 2 cohort studies focusing on safe elective operating during a period of low population incidence of viral infection. We report the experience of the London Collaborative throughout the first wave of the pandemic during the peak of infection and resource limitation.

**Added value of this study**

Our data demonstrate that safe elective thoracic surgery can be maintained during viral pandemic peaks in areas of high SARS-CoV-2 infection provided strict screening and isolation protocols are maintained. Furthermore, minimal access surgery (both thoracoscopic and robotic assisted) remains safe and should be continued where possible.

**Implication of all the available evidence**

We provide a framework for elective operating during the pandemic through qualitative descriptions of experience and consensus opinion across a global city. Many surgical units have struggled to maintain elective case volume, whilst oncologists have emphasised the impact of treatment delay on both stage migration and survival. This data highlights the need for collaboration to optimise treatment pathways, learning from the experiences of other regional centres to provide evidence based, safe, and timely radical surgery for lung cancer.

Alt-text: Unlabelled box

## 1 Introduction

In December 2019, an outbreak of atypical pneumonia was first reported in Wuhan, China. A novel respiratory coronavirus, named SARS-CoV-2 (severe acute respiratory syndrome-coronavirus-2) [1] spread globally and as of the May 2021 there have been more than 173 million confirmed cases of SARS-CoV-2 infection including more than 3.7 million deaths [2].

Within London, the first case of SARS-CoV-2 infection was confirmed on the 11th of February 2020 and there have subsequently been 721, 953 confirmed cases with 19, 083 deaths with SARS-CoV-2 on the death certificate in a population of almost 9 million. On the 23rd of March, the UK government imposed a national lockdown. At the peak of the first wave on the 2nd of April, more than 5000 hospital beds were occupied by patients with confirmed SARS-CoV-2 infection representing nearly a quarter of all inpatient bed capacity. At this peak, there were 1073 new confirmed cases within a single 24-h period [3]. In response to the increasing demand for intensive care (ITU) beds, unprecedented measures were implemented including the deferral of thousands of non-urgent operations, the use of private hospitals by NHS patients and the creation of new sites such as the Nightingale Hospital.

In the aftermath of these changes, many cancer clinicians have considered the ramifications of delaying or deferring cancer care. Some estimates are alarming, with current estimates of thousands of additional cancer-related deaths and tens of thousands of years of life lost [4]. The reasons for deferral are logical, both chemotherapy and surgery have demonstrable immunosuppressive effects and some hypothesised that radiation pneumonitis could potentially increase the severity of SARS-CoV-2 infection. However, more recently data has begun to emerge that cancer therapy including chemotherapy can continue without a significant increase in adverse effects during the pandemic [5]. From a surgical perspective, initial research from China demonstrated a 20% mortality rate in patients undergoing surgery who developed SARS-CoV-2 infection [6]. More recent data from Dublin, however, suggested that lung resection could proceed with appropriate safety measures [7].

In order to provide a forum for the dissemination of data regarding lung cancer patients in the high volume setting of a global city, we created a collaborative group including all thoracic surgical centres in London. We aimed to investigate the outcomes of anatomical lung resection in a general population with high rates of SARS-CoV-2 infection, better guiding future oncological decision making and demonstrating whether it is possible to safely maintain high surgical volumes despite the current obstacles to care.

## 2 Methods

**Fig. 1:**
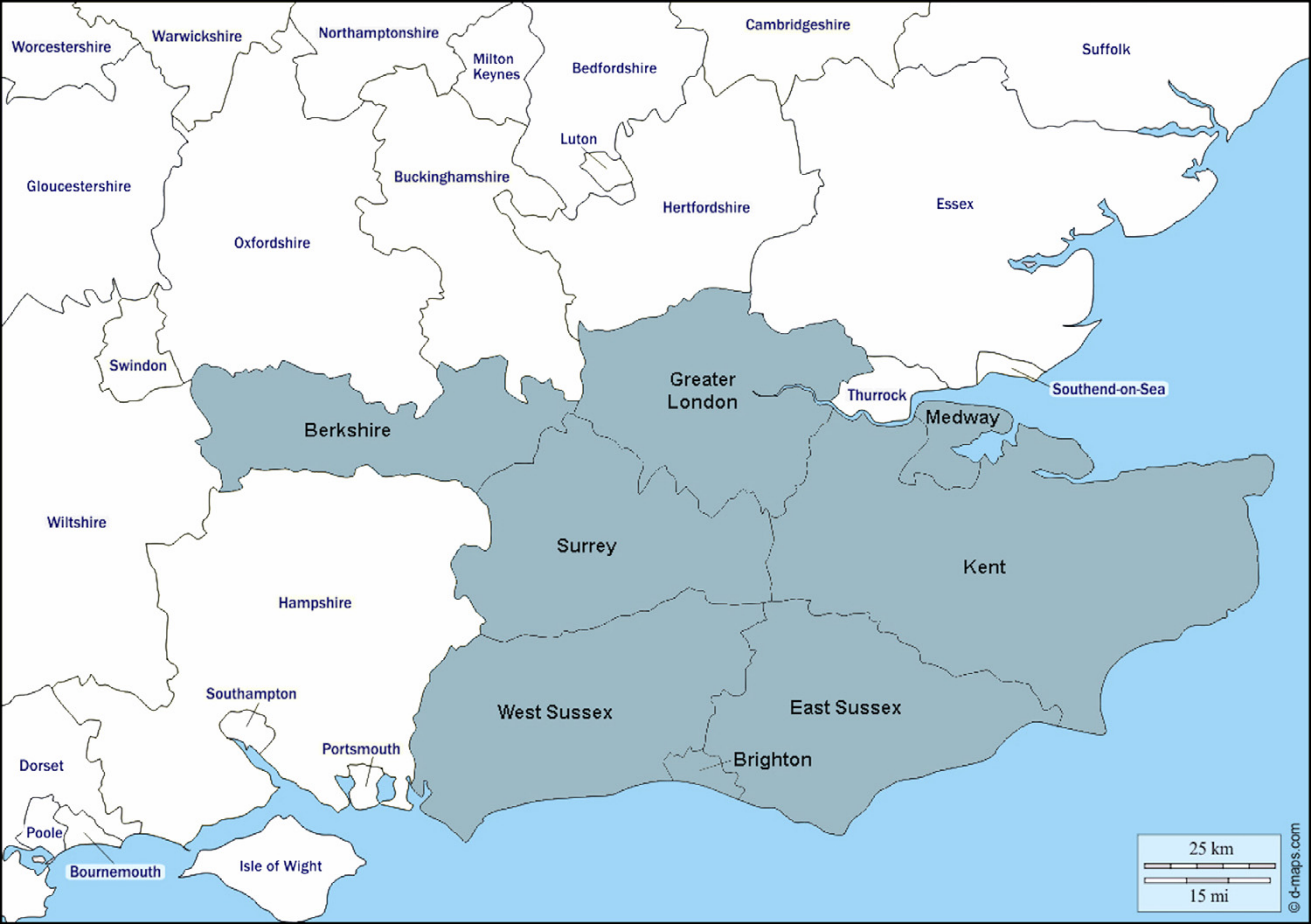
Catchment area of London Thoracic Surgical Centres. Dark grey area denotes regions covered by the pan London collaborative.

The Pan-London Thoracic Collaborative was set up in June 2020 in response to the SARS-CoV-2 pandemic. All thoracic surgical centres across London agreed to participate in the initiative. The referral base for these centres expands across Southern English Counties encompassing an estimated overall population of 14.8 million and accounting for approximately 22% of the entire UK population (Fig. 1) [8]. Institutional approval was obtained from all Trusts contributing to the collaborative. Approval was granted by each hospital trust for data sharing, collaborative work and retrospective review. Formal individual informed consent was not required due to the retrospective nature of the study.

The inclusion criterion for this study was all anatomical lung resections performed between 1st March and 1st June 2020 (inclusive). Non-anatomical lung resections and other thoracic surgical procedures were excluded. There was no age restriction. Demographic, clinical and survival data for those patients who fulfilled the inclusion criteria were collected retrospectively from review of case records. The primary outcome was death during follow up. Secondary outcomes were post-operative complications by Clavien-Dindo classification and diagnosis of SARS-CoV-2 infection. Referring units were also required to submit information on the type and timing of SARS-CoV-2 protocols within their institution including any screening methodology put in place and any other strategies to mitigate SARS- CoV-2 infection risk. Analysis was performed on an intention to treat basis meaning that although the majority of patients had a confirmed cancer diagnosis, suspected cancers found to be benign or metastatic on final pathology were included in the results.

Data was collected from all 7 London Thoracic Surgical Units. Surgery was performed at a total of 9 hospital sites, owing to the development of ‘COVID free’ beds at nearby hospitals. The practice of pre-operative screening and isolation of patients was implemented in line with trust protocols on each site with no change to standard operating procedures occurring prior to *26th* March 2020. Self-isolation was not mandatory at all units, however, in some units patients that chose to self-isolate were placed on a ‘Green’ protected pathway. This pathway was defined as admission only to areas of the hospital where all other patients had completed self-isolation and had a negative screening swab prior to admission. Other institutions were able to admit all patients to side rooms until pre-operative swab results were available. A summary of these protocols is shown in Table 1.

**Table 1:** SARS-CoV-2 specific admission and isolation protocols.

Site	Admission	Isolation	Screening
Guy's and St Thomas’ Hospital	No elective operating after 28/03/20 Prior to 28/03/20 Standard admission and operating procedures with no SARS-CoV-2 specific screening		
Hammersmith Hospital	No elective operating after 31/03/20 Prior to 31/03/20 Standard admission and operating procedures with no SARS-CoV-2 specific screening		
Harefield Hospital	48 h pre-operatively Date Implemented: 26/03/20	14 days pre-operatively, strict self-isolation Date implemented: 26/03/20	CT and nasopharangeal swab 48 h pre-operatively Date Implemented: 26/03/20
London Bridge Hospital	24 h pre-operatively (48 hrs at weekends) Date implemented: 28/03/20	14 days pre-operatively, strict self-isolation Date implemented: 28/03/20	Naso-pharyngeal swab on admission Date implemented: 28/03/20
Royal Brompton Hospital	No elective operating after 26/03/20 Prior to 26/03/20 Standard admission and operating procedures with no SARS-CoV-2 specific screening		
Royal Marsden Hospital	24 h pre-operatively (48 h at weekends) Date implemented: 26/03/20	14 days pre-operatively, strict self-isolation Date implemented: 26/03/20	Naso-pharyngeal swab and chest CT on admission Date implemented: 26/03/20
St Bartholomew's Hospital	48 h pre-operatively Date implemented: 26/03/20	Advised 14 days pre-operative self-isolation although not mandatory Date implemented: 26/03/20	Nose/throat swab on admission, trachea-bronchial swab in theatre, side room until results of deep swab available Date implemented: 26/03/20
St George's Hospital	Patients could be admitted on day of surgery or 24 h prior as per clinical requirement Unchanged from usual clinical practice	Non-mandatory pre-operative isolation, patients who chose to isolate were placed in protected pathway, Date implemented: 04/05/20	Swabbing 72 h pre-operatively Date implemented: 29/03/20
University College London Hospital	48 h pre-operatively Date implemented: 01/03/20	No pre-operative isolation	Nose/throat swab on admission, side room until results of deep swab available Date implemented: 26/03/20

Continuous variables were assessed for normality using Shapiro-Wilk testing. Non normally distributed variables were expressed as median and interquartile range (IQR), normally distributed variables expressed as mean ± standard deviation. Categorical data were expressed as counts and percentages. Univariable logistic analysis was performed to explore whether any patient and procedure-related factors were associated with higher risk of any complication and death in our cohort. Statistical significance was defined where *p*<0.05. All analysis was carried out using STATA version 12.0 (Leanne Harling, Imperial College London).

### 2.1 Role of funding source

This work received no specific funding.

## 3 Results

**Fig. 2:**
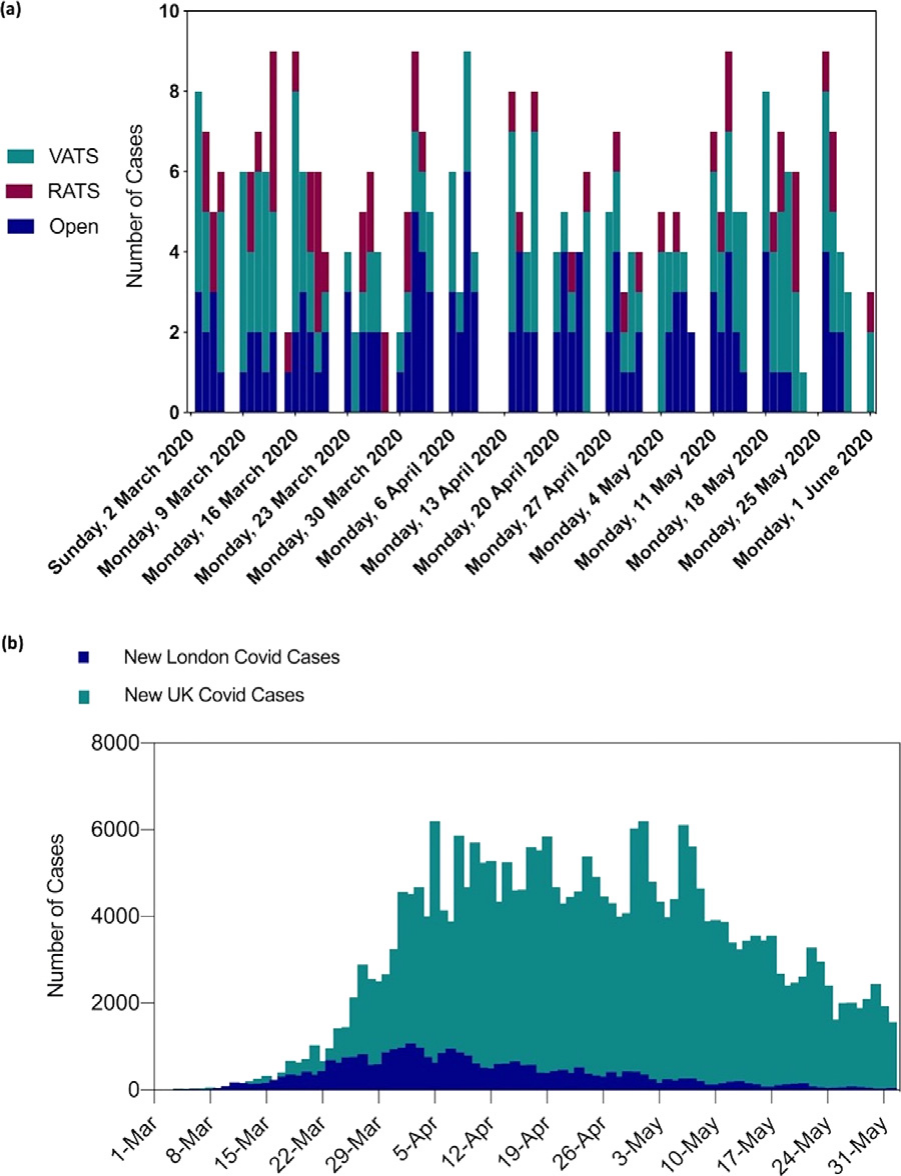
(a) Lung resections by surgical approach; (b) SARS-CoV-2 cases. (VATS: Video Assisted Thoracic Surgery; RATS: Robotic Assisted Thoracic Surgery).

The breakdown of cases performed at each site is shown in Table 2. A total of 352 anatomical lung resections were performed during the study period. This compares favourably to the 521 cases performed during the same three months in 2019 (Table 2). The breakdown of resection types is shown in Table 3. Initial concerns regarding the role of minimally invasive surgery (MIS) during the pandemic [9] relating to CO2 insufflation used for robotic procedures led to an early move towards open surgery (Fig. 2a). However, on consultation with virology teams, our SOP was quickly adapted to reflect the fact that MIS lung resection in the thorax can be performed without excessive aerosolization. As such, the majority of cases were performed via video-assisted thoracoscopic surgery (VATS; 45.5%), with 38.4% being performed via an open thoracotomy and 16.2% robotic-assisted thoracoscopic surgery (RATS)(Table 3). Notably, the proportion of patients undergoing MIS reduced during the peak of the pandemic in London (between late March and mid-April) (Fig. 2). However the number of MIS procedures remained overall comparable to our baseline dataset taken from the most recent UK Lung Cancer Clinical Outcomes Publication (LCCOP) validated dataset of the same institutions (Table 4). The remainder of patient characteristics are shown in Table 3. 109 (31%) patients received no pre-operative screening for SARS-Cov2 (either swab or CT imaging). Notably all of these patients underwent surgery before 14th April 2020 when availability of PCR testing was extremely limited. 160 patients had CT imaging pre-operatively to assess for the presence of pulmonary infiltrates. 6 patients received pre-operative chemotherapy. There were 14 conversions from MIS to open approaches, all of which were controlled.

**Table 2:** Numbers of cases at each operative site.

Site	1stMarch-1stJune 2020		1stMarch-1stJune 2019	
	n	%	N	%
Guy's and St Thomas’ Hospital	42	11.9	160	30.7
Hammersmith Hospital	6	1.7	46	8.8
Harefield Hospital	29	8.2	75	14.4
London Bridge Hospital	74	21.0	0	0
Royal Brompton Hospital	4	1.1	58	11.1
Royal Marsden Hospital	39	11.1	0	0
St Bartholomew's Hospital	73	20.7	65	12.5
St George's Hospital	27	7.7	44	8.4
University College London Hospital (UCLH)	58	16.5	73	14.0
**TOTAL**	**352**		**521**

**Table 3:** Patient and procedural demographics.

	Study population*N* = 352
**Demographics**	**Median (IQR)**
Age	69 (19–92)
BMI	26.3 (14.8–45)
	**n/%**
Sex (M)	134 (38.1%)
Smoking	
Non-smoker	76 (21.7%)
Ex-smoker	103 (29.4%)
Current Smoker	171 (38.9%)
Ethnicity (*n* = 223)	
White Caucasian	208 (93.3%)
Hypertension (*n* = 350)	133 (38.0%)
Diabetes mellitus (*n* = 350)	50 (14.3%)
Ischaemic heart disease	32 (9.0%)
Chronic kidney disease	9 (2.6%)
Cerebrovascular disease (*n* = 350)	29 (8.3%)
COPD (*n* = 346)	94 (27.2%)
ACE-inhibitor (*n* = 341)	88 (25.6%)
Steroids	15 (14.4%)
Metformin	33 (9.7%)
Statin	130 (38.1%)
**Procedure**	
Lobectomy/Bilobectomy/sleeve	277 (78.7%)
Segmentectomy/Sublobar	67 (19.0%)
Pneumonectomy	2 (0.6%)
Lung resection + chest wall	4 (1.1%)
**Approach**	
Open	135 (38.4%)
VATS	160 (45.4%)
RATS	57 (16.2%)
**Pathology**	
Primary Lung Cancer	307 (87.5%)
Pulmonary Metastasis	27 (7.7%)
Benign	17 (4.8%)
**Pathological Stage**	
I	211 (60.0%)
IIA	19 (5.4%)
IIB	38 (10.8%)
IIIA	30 (8.5%)
IIIB	10 (2.8%)

**Table 4:** Population comparison with 2018 UK National Lung Cancer Audit dataset.

	Study populationMarch–June 2020*N* = 352	Baseline populationJanuary–December 2018*N* = 1832
**Procedure**		
Lobectomy/Bilobectomy/sleeve	277 (78.7%)	1399 (76%)
Segmentectomy/Sublobar	67 (19.0%)	330 (18%)
Pneumonectomy	2 (0.6%)	40 (2.2%)
Lung resection + chest wall	4 (1.1%)	37 (2.0%)
**Approach**		
Open	135 (38.4%)	724 (40.0%)
VATS	160 (45.4%)	988 (54.6%)
RATS	57 (16.2%)	97 (5.3%)
**Outcomes**		
Length of Stay (Median(IQR))	6 (2–33)	6 (4–9)
30-day Survival	98.3%	98.4%

Results reported as n(%) or Median(IQR).

Table 5 displays the outcomes after surgery. 101 (28.6%) patients went to ICU post operatively, for a median of 1 day (IQR: 1–8). The rate of ICU admission was higher than pre-pandemic where only 7.6%* of patients were admitted to ICU (*compared to a representative sample of 158 patients undergoing lung resection 1st March to 1st June 2018). This largely reflects the standard operating procedures of the private hospitals which were free of any SARS-CoV-2 infection and where ICUs served as overnight recovery. 9/352 (2.6%) patients received non-invasive ventilation and 3/352 (0.8%) patients required invasive ventilation post operatively. Overall hospital stay was a median of 6 days (IQR: 2–33), with length of stay from operation to discharge a median of 4 days (IQR: 1–26). Patients were followed up for a median of 328 days (IQR: 299–351). Complications were seen in 162/352 (45.7%) patients. 130/352 (36.9%) were Clavien-Dindo grade 1–2 comprising of hospital acquired chest infection (HAP), post-operative atrial fibrillation (POAF), and prolonged air leak (or a combination of these). More severe complications (Clavien-Dindo grade 3–4) occurred in 26/352 (7.3%) patients. Return to theatre occurred in 12/352 (3.4%) patients and included 6 cases of bleeding, 3 bronchoscopy and bronchial toilet, 2 surgical repair of bronchopleural fistulae and 1 return to theatre for completion lobectomy (due to infection). The remainder of severe complications were treated supportively on the ICU with renal replacement (*n* = 3), vasopressor support (*n* = 3), and antibiotic treatment of severe chest sepsis requiring NIV (*n* = 9). There were 12/352 (3.4%) readmissions following discharge, at a median of 11(8–13) days. Five of these were due to persistent air leak or pneumothorax. Death during follow up occurred in 6/352 (1.7%) patients at a median of 19 days (IQR: 5–24) following surgery. Supplementary Table 1 displays the characteristics of patients who died. Cause of death was respiratory failure secondary to SARS-CoV-2 in 2 cases, in the remaining 4 cases cause of death was arrhythmia, self-harm, and pulmonary embolus. In one case death occurred at home without defined cause. Notably, length of stay and overall survival remained comparable to baseline data (Table 4).

**Table 5:** Outcomes after surgery (*n* = 352).

	n/%
ICU admission Post-op admission Non-invasive ventilation Invasive ventilation	101 (28.6%) 9 (2.6%) 3 (0.8%)
Complications Any complication Clavien-Dindo grade 1–2 Clavien-Dindo grade 3–4 Return to theatre	162 (45.7%) 130 (36.9%) 26 (7.3%) 12 (3.4%)
*Re*-admission	12 (3.4%)
Death	6 (1.7%)

Seven (2.0%) patients were diagnosed as positive for SARS-COV-2 at a median of 4 days post operatively (4–28) (Table 6). Notably, one patient tested positive pre-operatively and had surgery delayed for 4 weeks until clinically well and a negative swab result was confirmed. Of the remaining 6 post-operative SARS-COV-2 positive cases, only one was diagnosed after implementation of SARS-COV-2 specific admission, isolation and screening protocols. Two patients died during the post-operative period, both were diagnosed with coronavirus disease post operatively. The first death (Patient 1) was a white Caucasian 61-year-old male admitted electively for lung and rib resection for a cT3N0 biopsy proven adenocarcinoma. His-only significant past medical history was hypertension, his body mass index was 26.1. His-operation occurred at the beginning of the pandemic in London, prior to the implementation of lockdown measures. No pre-operative swabbing for SARS-COV-2 infection or CT screening was performed. His-recovery was initially uncomplicated however he became pyrexial and tested positive for SARS-COV-2 infection on day 4 post-operatively. He recovered sufficiently to be discharged on day 6 with some ongoing pyrexia with advice from the local SARS-CoV-2 medical and infection control teams. He was readmitted to his local hospital intensive care unit 5 days after his discharge with multi-organ failure and died on day 17 post-operatively.

**Table 6:** Characteristics and outcome in Patients with SARS-COV-2 infection.

	Patient 1	Patient 2	Patient 3	Patient 4	Patient 5	Patient 6	Patient 7
Age	**61**	**81**	65	70	82	79	75
Sex	**M**	**M**	M	M	F	F	F
BMI (kg/m2)	**25.0**	**27.4**	30.6	31.2	20.3	37.0	24.4
Smoking (Ex; Non; Current)	**Non**	**Ex**	Ex	Ex	Non	Ex	Ex
Ethnicity (WC; non-WC; Miss)	**WC**	**WC**	WC	WC	Miss	Miss	WC
**Medical history** Hypertension Diabetes Ischaemic Heart Disease Chronic Kidney Disease Cerebrovascular Disease COPD	**Yes** **No** **No** **No** **No** **No**	**Yes** **Yes** **No** **No** **No** **No**	Yes No No No No No	Yes No No No No No	Yes No No Yes No No	Yes No No No No Yes	Yes No No No No No
**Pre-operative medications** ACE-Inhibitor use Steroid use pre-operatively Metformin use Statin use Pre-operative chemotherapy	**No** **No** **No** **No** **No**	**No** **No** **No** **Yes** **No**	Yes No No Yes No	No No No No No	Yes No No Yes No	No No No No No	No Yes No No No
Pre-operative CT	**No**	**No**	No	Missing	PET	No	No
**SARS-COV-2 infection** Positive swab – days since Op Negative pre-op swab	**4** **N/A**	**2** **Yes**	4 N/A	−28 N/A	0 Yes	28 N/A	29 N/A
**Operation** Month of Operation Surgical approach Procedure Conversion In-hospital length of stay	**March** **Open** **Lobect+cw** **No** **8 days**	**April** **Open** **Lobect** **No** **RIP Day 4**	March Open Lobect Yes 10 days	May Open Lobect No 5 days	June RATS Other No 6 days	March RATS Lobect No 12 days	March VATS Lobect No 15 days
**Outcomes** Complications Calvien Dindo complication grade Hospital re-admission Mortality	**Yes** **5** **Yes** **Yes**	**Yes** **5** **No** **Yes**	Yes 2 No No	No 0 No No	No 0 No No	Yes 2 No No	Yes 2 Yes No

*died during admission.

WC: White Caucasian; EX: Ex-smoker; Non: Non-Smoker.

VATS: Video-assisted thoracoscopic surgery; RATS: Robotic-assisted thoracoscopic surgery.

Lobect: Lobectomy/Bilobectomy; Segem: Segmentectomy.

Cw: chest wall.

The second death (Patient 2) was a white Caucasian 80-year-old male whose initial pre-operative naso-pharyngeal swab was negative for SARS-COV-2 infection. His-past medical history included hypertension, diabetes mellitus, hypercholesterolaemia and prostate cancer. He underwent an open lobectomy for a pT2aN2 R0 adenocarcinoma which had a pre-operative staging of T2aN0M0 . His-procedure was uncomplicated, however he developed early signs of respiratory failure post-operatively and was managed in the high dependency unit followed by ITU. Thorough clinical and radiological investigation at this point was entirely consistent with SARS-CoV-2, and despite a further negative swab multidisciplinary consensus view was of SARS-CoV-2 infection. He was treated with non-invasive ventilation and antiviral therapy however, continued to deteriorate and died on day 4 post-operatively from respiratory failure.

Patient and procedure-related factors for any complication and death were assessed via univariable logistic regression (Supplementary Table 2). As expected, male sex and higher risk surgical procedures including sleeve lobectomy and chest wall resections when compared to lobectomy were significant risk factors for ‘any’ complication and death, whilst hypertension, COPD and an open approach were risk factors for ‘any’ complication.

Multivariable regression has been performed using 2 logistic models. Model 1 included SARS-CoV-2 infection, age, gender BMI and procedure type. Owing to collinearity between procedure type and the use of minimal access approach, a second model was built utilising the latter variable. SARS-COV-2 infection remained the most significant predictor of death in both models (Model 1: OR 17.2; *p* = 0.008; Model 2: OR 20.1; *p* = 0.007) however procedure other than lobectomy/segmentectomy was also associated with significantly higher mortality in model 1 (OR 9.0; *p* = 0.043)(Supplementary Table 3a). When examining the all complications outcome, male gender, procedure other than lobectomy/segmentectomy (Model 1), and open approach (Model 2), were associated with significantly higher all cause complications (Supplementary Table 3b).

## 4 Discussion

This is the largest UK study to date of thoracic surgical patients undergoing treatment during the coronavirus pandemic based in the global city of London. We demonstrate the safety and tolerability of anatomical lung resection during the initial peak with parity of access to minimally-invasive surgery. Patients undergoing thoracic surgery across London had similar morbidity and mortality rates as those from the pre-SARS-CoV-2 era, the average length of stay was comparable to previous data and re-admission rates were lower than previously reported [10].

We have demonstrated the importance of collaborative work during periods of unprecedented change. The Pan-London Thoracic Collaborative provided a forum to discuss changes to standard operating practice across NHS Trusts and helped units to rapidly adapt their practice alongside evolving evidence. There was clear geographical variation in the number of SARS-CoV-2 cases across London. The corresponding variation in activity between units partly reflects this discrepancy, but was also influenced by factors such as the number of ITU beds, the number of beds occupied by patients with SARS-COV-2 infection, the presence of a large infectious disease team, the availability of extra-corporeal membrane oxygenation and the availability of a separate ‘clean’ site. These institutional differences highlight the importance of pooling resources and offering surgeons honorary contracts at other sites across London to reduce inequalities in cancer care.

These data contribute to the ongoing discussion regarding optimal timing of lung resection. Initial evidence in the coronavirus pandemic supported the deferral or delay of surgery in ‘stable cancer’ [11]. Guidelines from the American College of Surgeons suggested a surgical delay for tumours such as ground-glass nodules where survivorship without radical treatment was felt to be possible with a three-month deferral [12]. However, we propose that a delay in radical surgical treatment for any early stage lung cancer, due to the fear of worse outcome relating to SARS-CoV-2 infection, is not indicated if appropriate measures are taken to protect patients during the peri-operative period. The low incidence of severe (Clavien-Dindo grade 3+) morbidity and mortality observed in our patient group suggests that operating should continue at the current volume, even in the instance of a second or subsequent wave of SARS-CoV-2 infections.

Our data support previous literature which highlighted the increased risks of SARS-CoV-2 infection in lung cancer patients [13]. Of the seven patients who developed SARS-CoV-2 infection, there was a 28.5% mortality rate (*n* = 2), compared to a 15% fatality rate in the general populace of the UK [14]. Some research has suggested that surgery could accelerate the disease process [6] while others reported that patient-specific factors such as age and co-morbidity were independent risk factors for poor outcome rather than cancer-specific factors such as previous surgery [15]. Either way, measures to reduce the risk of SARS-CoV-2 infection in patients awaiting cancer treatment is clearly the goal for surgical teams.

It is our consensus opinion that surgical treatment should be deferred when there is a SARS-CoV-2 diagnosis pre-operatively. We have successfully operated on patients who tested positive on pre-operative naso-pharyngeal swabbing after an interval period of one month with no unanticipated morbidity or longer term sequalae. The optimum timing of surgery following SARS-CoV-2 infection should be decided in conjunction with local infection control and virology teams, but at least a four week delay is supported by international data from the COVIDSurg Collaborative  [16].

During the study period, advice was implemented in England which suggested a 14-day period of mandatory self-isolation and SARS-CoV-2 naso-pharyngeal swabbing within 72 h for all asymptomatic patients undergoing elective surgery [17]. From our data, the implementation of mandatory self-isolation had a significant positive impact on patient outcomes, with no cases of coronavirus identified during the in-patient stay after this was in effect.

Recent NICE guidance has suggested a move towards eleven days of social-distancing and careful hand hygiene, with strict isolation for only three days prior to surgery [18]. However, in our experience of the second wave this led to outbreaks of SARS-CoV-2 infection and we continue to advocate a 14-day mandatory self-isolation period during the peak of a pandemic when community rates of infection are high. We also advocate that patients are encouraged to continue shielding post-operatively during pandemic peaks. This relates to recent literature which studied the higher rates of SARS-CoV-2 infection in patients with non-small cell lung cancer (NSCLC) and found that surgery within the last month was a risk factor for severe morbidity and mortality [19].

As per recent literature from the COVIDSURG Collaborative, pre-operative nose and throat PCR swabbing is effective in reducing pulmonary complications associated with major surgery. Routine swabbing was introduced a third of the way through our study period across London and therefore early patients who developed SARS-CoV-2 infection were not screened. The low volume of SARS-CoV-2 infection in our population despite the high rates of infection in London during this time reflect the robustness of pre-operative swabbing and it is our group consensus that this would be an ideal SOP for maintaining safe surgery [20]. Our data also however re-emphasises the fact that false negative results from pre-operative nose and throat SARS-CoV-2 RT-PCR swabbing can occur [21]. The impact of inaccurate swabbing is highlighted in the case we present of a patient with a clinical and radiological diagnosis of SARS-CoV-2 but negative pre- and post-operative coronavirus swab results, who ultimately died as a result of his SARS-CoV-2 infection in the early post-operative period. Following our move back to NHS hospitals with ‘protected’ and ‘non-protected’ pathways and fewer side rooms, it was essential that the sensitivity of swabbing was assessed and that an effective self-swabbing protocols were implemented to improve safety for both patients and healthcare professionals.

A published consensus opinion of individuals involved in managing thoracic malignancies supported the use of pre-operative CT screening for SARS-CoV-2 [22]. At the onset of the pandemic, owing to the limited testing capacity and initial concerns regarding the accuracy of nose and throat PCR swabbing, CT screening was utilised at several London Centres. This was not however supported by subsequent research from the COVIDSurg Collaborative or the UK consensus of the combined Royal Colleges and Associations of Surgeons [17,20]. Their data highlights the low sensitivity and specificity of a pre-operative CT chest in diagnosing SARS-CoV-2 infection in asymptomatic patients prior to elective surgery. They do not recommend it as a suitable screening tool, but advise that it can be employed in certain circumstances such as patients who are planned to recover in HDU or ITU areas [23]. In practical terms, the need for admission 1.5–2 days prior to surgery for naso-pharnygeal swabbing followed by CT scanning and then reporting, limited the use of this protocol, particularly when beds were in short supply. From our data however, the lack of pre-operative CT screening was an independent risk factor for SARS-CoV-2 infection.

The results of this study should be considered in the context of a number of limitations. First, we appreciate at present there is a lack of long-term follow up to determine the sequelae of SARS-CoV-2 infection in thoracic surgical patients. Second, clinical stage data were limited, resulting in difficulties commenting on stage progression. This is in part due to the impact of the pandemic on aerosol generating procedures such as endobronchial ultrasound (EBUS), which were completely suspended in a number of peripheral referral centres. It is therefore essential that we continue to study the oncological outcomes of patients treated during the coronavirus pandemic and strive to overcome these limitations for future waves. Many current oncology models suggest that priority setting could result in either delays in treatment, or the decision not to proceed with adjuvant chemotherapy for NSCLC following surgery [24]. Within our own cohort, at least one patient did not receive adjuvant treatment following a referral to oncology due to the coronavirus pandemic. The patient was found to have disease progression at 3-month surveillance imaging, which was subsequently proven histologically. Whilst we appreciate surgical intervention itself confers risks of major morbidity and mortality, the overall mortality from surgery remains low in comparison to the reduction in survival in patients receiving delayed or no treatment [25].

As part of shared decision making, all patients with early stage disease were counselled regarding their options for radical treatment including surgery and sterotactic radiotherapy (SBRT). There is also evidence for upfront SBRT followed by a salvage lobectomy to avoid the peri-operative risks of SARS-CoV-2 infection [26]. However, it was our experience at the initial peak of the pandemic that access to SBRT was more limited than usual, in part due to concerns regarding viral pneumonitis. All options should be discussed with patients as part of routine counselling with the aim of ensuring radical treatment for the largest proportion of early stage lung cancer patients.

We also must consider bias such as low re-admission rates reflecting the inability of patients to safely access primary care or local hospital follow up rather than a true improvement in outcomes. Current rates of 90-day re-admission in the UK following lung resection are approximately 41% [10], but with reduced access to general practitioners (GPs) and district nurses, post-operative complications such as pain and respiratory tract infections may not be identified or appropriately managed.

The route by which new diagnoses of lung cancer present must also be considered as we move forward. Prior to the coronavirus pandemic, around 41% of new diagnoses of lung cancer were made following a GP referral. During the coronavirus pandemic, the overlap of respiratory symptoms relating to lung cancer and SARS-CoV-2 infection resulted in many patients in the UK being advised to self-isolate rather than present to their GP [27]. This is consistent with data which demonstrated up to an 80% reduction in urgent referrals for suspected cancer [4]. This is echoed in other oncology work which demonstrated that a two-month delay in urgent referral for investigation results in up to 0.7 life-years lost per patient and that young cancer patients were disproportionately affected by delays to their care [28]. We must consider ways of identifying this cohort of patients. Although some recent literature suggested that lung cancer screening and nodule follow up should be deferred [29], the benefits and risks of wider enrolment in lung cancer CT screening programmes should be considered as a means of reducing this inequality in cancer care.

SARS-CoV-2 infection results in significantly increased morbidity and mortality in patients undergoing elective anatomical pulmonary resection for primary lung cancer. However, elective anatomical pulmonary resection for primary lung cancer surgical resection remains feasible and safe during pandemic peaks provided strict admission, isolation and testing protocols are implemented. Treatment pathways should be maintained and unnecessary delays to treatment avoided, so as to prevent the unnecessary morbidity and mortality associated with disease progression.

Early establishment of collaborative groups to guide surgical speciality-specific decision making is of paramount importance to ensure rapid refinement of protocols and streamlining of care pathways. Furthermore, such collaborations facilitate the development of reporting systems and learning from a larger evidence base in the event of a rapidly evolving pandemic situation.

## Authors’ contributions

LH designed and oversaw the project. SF, RB, MH and HD provided additional project visualisation. LH, SF and HM designed the project methodology. Project administration and investigation was carried out by LH, SF, RB, JN, MAS, DP, JS and RH who acted as representatives for the institutions in the collaborative, alongside HD who provided support from the South East London Cancer Alliance. LH, SF, RB, DP, JN, MAS, RH, JN, GS, ML, AW, MJ, KHP, JK, JP, AB, LO, SS, DW, HW, SJ, S Begum, S Buderi, CT, IH, PV, MJ, MH, DL, EB, VA, AM, JF, TR, KL, EL, and LH all contributed to data curation. Data were validated by MAS, JS, RH, JN, DP, RB, LH and SF. KL, EL, TR and LH also contributed resources and supervision of the project. Formal analysis was performed by LH and HM. Writing of the original draft and revised manuscripts was performed by LH, SF, RB and HM.

## Data sharing statement

No additional unpublished data are available.

## Funding

This work received no specific funding

## Declaration of Competing Interest

None of the authors have conflict of interests to declare.
